# Progress in Gynæcology

**Published:** 1899-08-19

**Authors:** 


### PROGRESS IN GYNÆCOLOGY.

(*Continued from page* 333.)

**Gonorrhœa in Women.**—Eastman[5] says there is no malady to which womankind is liable that should engage more consideration than gonorrhœa, seeing the pathologic ravages of surpassing import that may follow this disease; and he points out that there exists a notable, if not notorious tendency on the part of most general practitioners and some specialists to disregard discriminatory diagnosis in doubtful cases. He maintains that the diagnosis of acute gonorrhœa in women is comparatively easy even without the microscope, the cardinal symptoms, free purulent secretion from vulva, vagina, and urethra, intertrigo, burning on micturition and vesical tenesmus being usually well marked. Upon inspection one usually detects a discharge of tenacious pus. The labia minora, the clitoris and its prepuce, and the hymen, if present, are red and swollen. The meatus urinarius is found to be congested and ectropic, its normal pink colour being changed to a deep red. The mouths of the ducts of the Bartholinian glands are deeper in colour, gaping, and tender. The surface of the vagina proper presents no great changes, the adult vaginal mucous membrane being practically uninfluenced by the presence of the gonococcus, not for the reason generally stated, viz., that the germ cannot exist upon flat epithelium, but more probably because, as Doederlein has pointed out, of an acid environment and the presence of the vaginal bacillus. The only gross pathological lesions detectable in the vagina are minute angry red spots emitting a drab-coloured secretion. After a time—usually about a week—the inflammation spreads to the uterus, and is here announced by symptoms of pain and tenderness in that organ, with sometimes fever and a purulent discharge from the cervix uteri. There is a difference of opinion as to the relative value of the microscope in diagnosing gonorrhœa in the female. Saenger and Broese ascribe to it very little worth, whilst Neisser attaches great importance to this method. A comprehensive examination of the discharge is not complete until the secretion of the urethra, Skene's læunæ, the glands of Bartholin, the vagina and cervix, have been searched through, and in chronic cases several preparations should be made from each of these. In preparations of acute gonorrhœa we expect to find large gonococci lying in groups within and without the cells. It is not to be assumed that because the organisms are intracellular that they are therefore gonococci, for it is certain that other diplococci than those of gonorrhœa lie within the cell protoplasm. Drobny[6] insists that the course of the disease depends on the intra or extra-cellular position of the gonococci. He thinks it possible that the extra-cellular position of the cocci may be due to paralysis of the leucocytes by toxins, thereby rendering them incapable of surrounding the cocci. In preparations from chronic urethritis of gonorrhœal origin, one finds epithelium in abundance, few pus cells, and sometimes gonococci; these usually appear, however, only after coitus, or after the application of an irritant to the mucosa. Greenberg,[7] writing on the treatment of gonorrhœal vaginitis, insists on the importance of active local treatment at the earliest possible moment. He says the gonococci in the vagina may be conquered with effective weapons; but when once they are entrenched and barricaded in the uterine canal, with a fertile soil to thrive on, our power of attack becomes limited or null. Rest in bed he considers indispensable in an acute attack. The diet should be light, and alcohol in any form strictly forbidden. Cleanliness is next in importance, as it affords greater resistance to the tissues attacked, and prevents the invaders from accumulating and multiplying. Sitz baths of warm water with sodium bicarbonate twice daily, with two-hourly douches of sodium bicarbonate solution, a gallon each time, will answer the purpose effectively. The sodium bicarbonate acts as a sedative rather than as a germicide. To kill the gonococci the vagina should be thoroughly scrubbed with soap and water, the tenacious and sticky pus from the cervix mechanically removed, and a 1-1,000 bichloride solution, or, better still, 5 per cent. methylene blue, thoroughly applied by means of cotton on an applicator. Dr. L. Fürst[8] strongly recommends largine for the treatment of gonorrhœa; he describes it as a greyish powder, easily soluble in water, forming a clear solution, which is feebly alkaline and quite stable if kept in coloured bottles. It is a silver compound, closely allied to protargol. He recommends an aqueous solution to be applied as an irrigant; it is well to begin with a weak solution—1-200—and increase the strength gradually. Cipriani[9] advocates the use of argentamine for the treatment of gonorrhœa in women; he uses a solution of 1-3,000 or 1-4,000, and says that in addition to its antiseptic properties, it has a powerful astringent action on the mucous membrane. The following astringent solution is recommended by J. D. Thomas[10]: ℞. Zinci sulph. alum sulph. a.a., ℥i.; cupri sulph. ferri sulph. a.a., ʒii. M.S. A teaspoonful to a pint of water, to be used as a vaginal douche. He also advises a local application of nitrate of silver to the vagina and cervix, 20-50 grains to the ounce to be made by the physician, twice a week. If the cervical canal is involved it may be treated with tincture of iodine and carbolic acid, equal parts of each, the application being made with a cottoned probe.

[5] Jour. of American Medical Association, Jan., 1899. [6] Brit. Med. Jour., Jan. 3, 1899. [7] New York Med. Jour., Mar. 25, 1899. [8] New York Med. Jour., May, 1899. [9] The Therapist, Feb. 15, 1899. [10] International Med. Mag., May, 1899.

